# A Hybrid Strategy for Patients With Complex Cerebral Aneurysm: STA–MCA Bypass in Combination With Endovascular Embolization

**DOI:** 10.3389/fneur.2020.614601

**Published:** 2021-01-15

**Authors:** Gang Wang, Xi'an Zhang, Yanxia Gou, Yunyu Wen, Guozhong Zhang, Mingzhou Li, Shichao Zhang, Yanyi Yin, Siyuan Chen, Songtao Qi, Wenfeng Feng

**Affiliations:** ^1^Department of Neurosurgery, Nanfang Hospital, Southern Medical University, Guangzhou, China; ^2^Department of Anesthesiology, Nanfang Hospital, Southern Medical University, Guangzhou, China

**Keywords:** cerebral complex aneurysm, hybrid surgery, superficial temporal artery-middle cerebral artery bypass, endovascular therapy, intraoperative evoked potential monitoring

## Abstract

**Objective:** This work aims to present our experience of patients with complex cerebral aneurysm treated with a hybrid approach: superficial temporal artery–middle cerebral artery (STA–MCA) bypass in combination with endovascular exclusion of the aneurysm.

**Method:** Patients with aneurysms deemed unclippable and uncoilable were included. All patients were treated with a hybrid approach. After STA–MCA bypass, the parent artery was temporarily occluded. If the intraoperative motor evoked potential (MEP) and somatosensory evoked potential (SEP) waveforms remain normal and last for 30 min, the aneurysm and te parent artery will be embolized permanently with detachable balloons or coils.

**Results:** A total of 20 patients with 22 aneurysms were included in this study. There were 13 women and 7 men, with an average age of 42.5 years. Intraoperative angiography showed the good patency of all the STA grafts, and neither SEP nor MEP abnormalities were detected. After the parent artery and the aneurysm were occluded, the intraoperative angiography showed an immediately successful exclusion of the aneurysm in 20 aneurysms and immediate contrast stasis in two. All patients recovered uneventfully without ischemic or hemorrhagic complication. Angiography at 6-month follow-up showed the total obliteration in 20 aneurysms. Two aneurysms showed residuals and were recoiled. All STA grafts showed a good patency, and the mean graft flow was 124.2 ml/min.

**Conclusion:** STA–MCA bypass in combination with endovascular exclusion is an appropriate option for patients with complex cerebral aneurysms that are not amenable to direct surgical clipping or endovascular embolization.

## Introduction

Complex cerebral aneurysms such as giant aneurysm, serpentine aneurysm, dissection aneurysm, and recurred aneurysm after embolization have always been and remain among the most difficult cerebrovascular lesions to treat, even for experienced neurovascular surgeons (1). Extracranial–intracranial (EC–IC) bypass with parent artery occlusion is one of the preferred procedures for complex aneurysms (2, 3). However, there are two debatable issues. First, the optimal bypass selection remains controversial (4–6). Besides that, the endovascular occlusion of the parent artery becomes more popular due to its less invasive nature (7).

Traditionally, the saphenous vein and the radial artery are selected as the graft for the high-flow bypass (8, 9), which has been proven as an effective and safe bypass. However, compared to the superficial temporal artery–middle cerebral artery (STA–MCA) bypass, the high-flow bypass (ECA–graft–ICA) required multiple incisions (head, neck, and arm or leg) and additional graft harvest, which made the procedure more invasive and technically difficult. STA was the first auto-graft used in EC–IC bypass and has been considered as low-flow graft. However, recent studies, including ours, have shown a significant increase in the mean flow rate of the STA graft after bypass surgery (10, 11). In Amin-Hanjani's study (12), the intraoperative cut flows of STA graft could exceed more than 100 ml/min in 16% cases, and Cherian et al. reported that 94% of the double-barrel STA–MCA bypasses had best observed flow >40 cc/min (13). More and more studies have revealed that the definition of a high-flow bypass should not be limited only to interposition grafts from cervical carotid branches (14). What is more, the concept of a flow replacement bypass does not mean replacing the entire territory of the occluded artery in virtue of the collateral flow coming from the communicating artery and/or the cortical arteries. Therefore, STA would be an efficient and simple graft for the treatment of complex aneurysm.

Here we present our experience in 20 patients with complex aneurysms treated with a hybrid approach: STA–MCA bypass in combination with endovascular exclusion of the aneurysm on the basis of preoperative multimodal imaging and intraoperative evoked potential monitoring.

## Patients and Methods

### Patients

This study was approved by the Institutional Review Board of Nanfang Hospital. Twenty patients with 22 complex aneurysms underwent hybrid surgery during the period from April 2015 to January 2020 in the Department of Neurosurgery, Nanfang Hospital, Southern Medical University. Complex intracranial aneurysms include those classified as giant, those that reoccurred after embolization treatment, or those that involve arterial trunks/branches. Their ages ranged from 8 to 60 years old, with an average age of 42.5 years. Thirteen patients were female, and the other seven were male. There were 21 unruptured aneurysms and one ruptured aneurysm. Two patients had multiple aneurysms in the same ICA territory. Seven aneurysms were located on the right side and 15 on the left side. Of the 22 aneurysms, there were seven giant saccular aneurysms, seven giant serpentine aneurysms, five recurrent aneurysms after embolization, and three dissection aneurysms. Fourteen aneurysms were found to be in the cavernous segment of the internal carotid artery, three were in the supraclinoid segment of ICA, two were in the M1 segment of the MCA, two were in the inferior trunk of the MCA, and one was in the superior trunk of the MCA (Table 1).

**Table 1:** Clinical characteristics of individuals with complex aneurysm treated by hybrid strategy.

**No**	**Sex**	**Age**	**Side**	**Aneurysm location**	**Aneurysm characteristic**	**BOT**	**Venous phase delay**	**Circle of Willis**	**Pre-operative GCS**	**STA bypass**	**Emboliazaiton strategy**	**Complication**	**Graft flow**	**GOS at FU**
1	Female	38	Right	ICA-C4	Giant, serpentine	Neg	Positive	A+P	15	Double	Balloons	None	NA	5
2	Male	49	Left	MCA-M2	Giant, serpentine	–	–	–	15	Single	Coils	None	NA	5
3	Female	48	Left	ICA-C4	BBA, recurrence	Neg	Positive	A	15	Double	Coils+balloons	None	NA	5
4	Male	49	Left	ICA-C3	Giant, serpentine	Neg	Positive	A	15	Double	Balloons	None	NA	5
5	Male	14	Left	ICA-C3	Giant, serpentine	Neg	Positive	A+P	15	Single	Balloons	None	181	5
6	Female	57	Left	ICA-C3	Giant, serpentine	Neg	Positive	A+P	15	Double	Balloons	None	73	5
7	Female	60	Right	ICA-C3	Giant, saccular	Neg	Positive	A	15	Double	Balloons	None	89	5
8^*^	Male	34	Left	ICA-C3	Giant, dissection	Neg	positive	A+P	15	Double	Coils+balloons	None	105	5
9	Female	22	Left	MCA-M1	Dissection, recurrence	–	–	–	15	Double	Coils	None	124	5
10	Male	8	Left	ICA-C3	Giant, serpentine	Neg	Positive	A+P	15	Double	Balloons	None	172	5
11^*^	Female	51	Left	ICA-C3 MCA-M1	Giant, saccular; dissection	Neg	Positive	A+P	15	Double	Coils+balloons	None	227	5
12	Male	39	Right	ICA-C3	Giant, saccular	Neg	Positive	A+P	15	Double	Balloons	None	NA	5
13	Female	55	Left	MCA-M2	Giant, recurrence	–	–	–	15	Double	Coils	None	66	5
14	Female	52	Right	ICA-C3	Giant, recurrence	Neg	Positive	A	15	Double	Balloons	None	NA	5
15	Female	56	Right	ICA-C4	Giant, saccular	Neg	Positive	P	8	Double	Balloons	None	177	3
16	Female	60	Right	ICA-C3	Giant, saccular	Neg	Positive	A	15	Double	Balloons	None	NA	5
17	Female	51	Left	ICA-C3	Giant, saccular	Neg	Positive	A+P	15	Double	Balloons	None	142	5
18	Male	35	Left	MCA-M2	Dissection, recurrence	–	–	–	15	Double	Colis	None	52	5
19	Female	50	Right	ICA-C3	Giant, saccular	Neg	Positive	A	15	Double	Balloons	None	NA	5
20	Female	22	Left	ICA-C3	Giant, serpentine	Neg	Positive	A+P	15	Double	Balloons	None	83	5

* *multiple aneurysms; ICA, internal carotid artery; MCA, middle cerebral aneurysm; C3, cavernous segment; neg, negative; A, anterior communicating artery; P, posterior communicating artery; NA, not available; GOS, Glasgow outcome scale; FU, follow up*.

### Imaging and Indications for Bypass Surgery

All patients were diagnosed with digital subtraction angiography and received a balloon occlusion test (BOT). The aneurysms in these patients were considered to be surgically unclippable based on digital subtraction angiography (DSA) findings and not suitable for endovascular embolization or the patient refused to receive a pipeline device due to economic reasons. The indication for STA–MCA bypass surgery was established based upon tolerance to BOT, which has been defined as neurologically intact during the 30-min parent vessel occlusion but any inadequate venous-phase delay. The anatomy of the circle of Willis was analyzed to confirm the presence of anterior communicating artery and/or ipsilateral posterior communicating artery, which make the scheduled bypass procedure a complementary bypass due to the existence of compensation flow from the contralateral or posterior circulation. The radial artery auto-graft was the first choice for a potential rescue bypass. The ipsilateral radial artery would be taken if the Allen test revealed an adequate blood supply to the palmar arcade and hand through the ulnar artery alone.

### Treatment Strategies and Surgical Techniques

**Figure 1:**
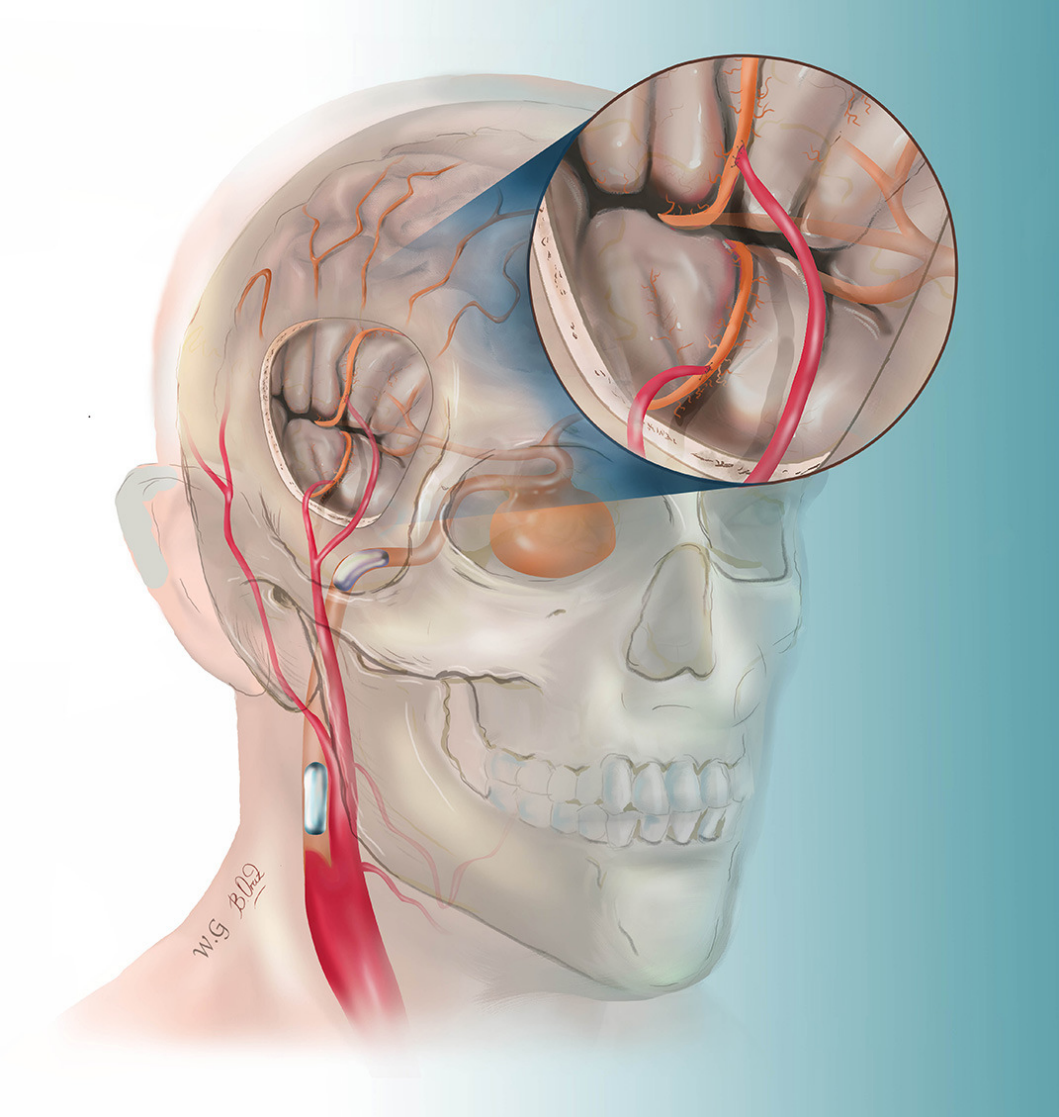
This drawing illustrates the hybrid strategy for complex internal carotid artery (ICA) aneurysm. After double superficial temporal artery–middle cerebral artery bypass, the ICA was permanently blocked by two detachable balloons.

The patients were treated in a hybrid operating room. The MEP and SEP were applied during the whole procedure. The targeted branches of the middle cerebral arteries were selected as recipient vessels. For ICA aneurysm, one frontal and one temporal cortical branch of the MCA were chosen as recipient arteries to get flexibility in distributing flow across separate territories. For MCA aneurysms, the exact efferent parent arteries were identified on the pre-surgical DSA with the assistance of double volume reconstruction. The projection of the target recipient arteries on the cranial bone was marked. The craniotomy was then performed based on the locations of the targeted recipient and donor vessels (STA branches). After the STA–MCA bypass was completed, intraoperative DSA was performed to verify the patency of the grafts. Then, endovascular temporary parent artery occlusion was used to observe the STA–MCA flow capacity. The evoked potentials were monitored. If the MEP or SEP waveforms decreased by 50% in amplitude compared with the baseline value, the balloon will be deflated, and in this case, a rescue bypass with radial artery graft will be performed. If the MEP and SEP waveforms remained normal for 30 min, the balloons were inflated and released to occlude the ICA (Figure 1). For MCA aneurysm, coils were used to embolize the aneurysm and parent artery.

Post-operative management included monitoring of the patients in the intensive care unit where arterial and central venous pressures were monitored. All the patients underwent CT, MR, DSA, and CT perfusion postoperatively. The blood flow (ml/min) of STA graft was measured by duplex ultrasonography and recorded. For single bypass, the checkpoint was located at the segment where the STA passes through the skull bone. For double-barrel bypass, the checkpoint was located just at 3–5 mm proximal to the bifurcation of the frontal and parietal branches of the STA (11).

## Result

**Figure 2:**
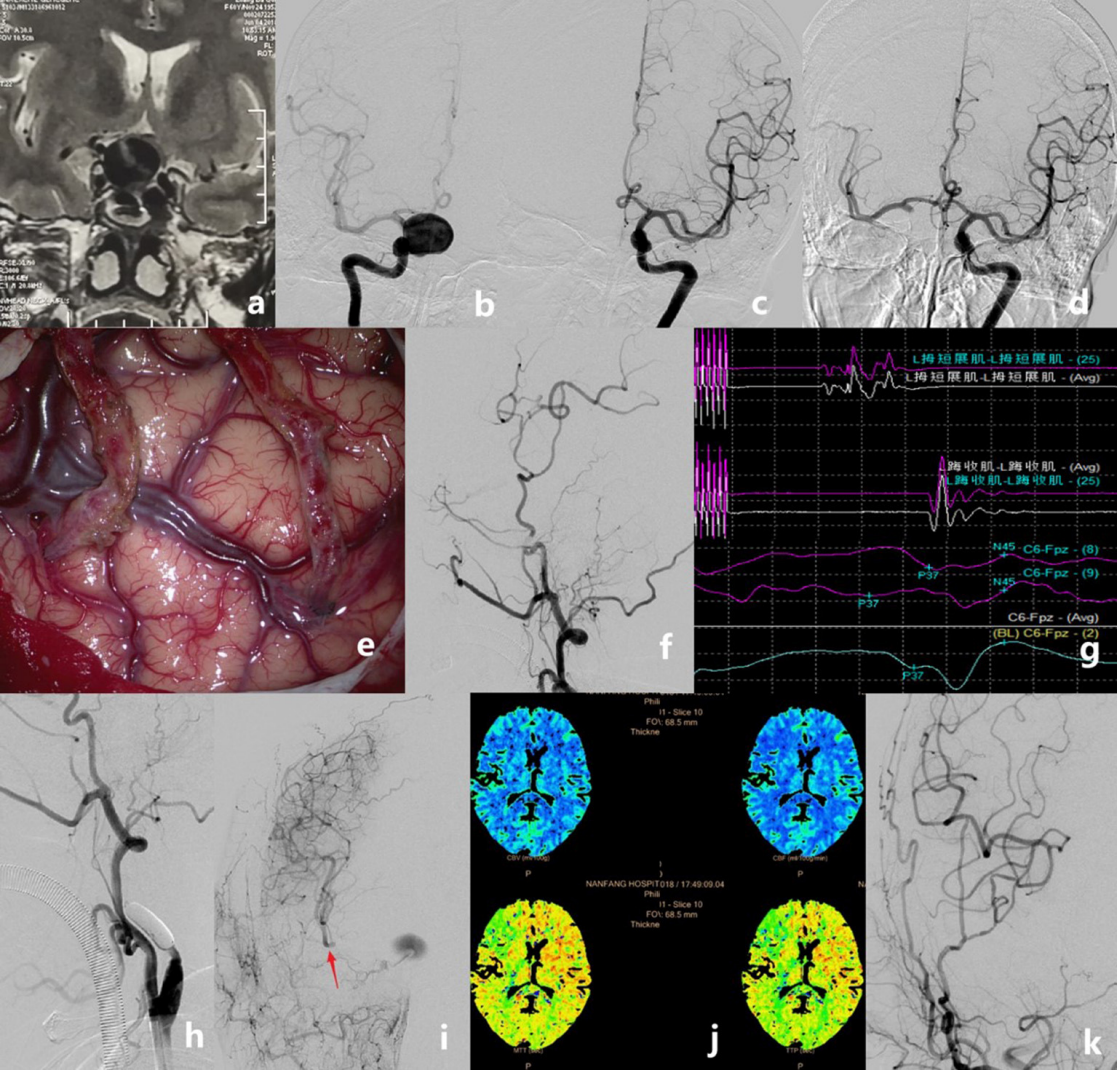
Case example of a patient who had a giant internal carotid artery (ICA) aneurysm. MRI showed a mass with fluid void effect in the sellar region **(a)**. Pre-operative angiograms showing that the aneurysm was located at the right C4 [**(b)**, anteroposterior view]. The anterior communicating artery did not show on regular left ICA angiogram [**(c)**, anteroposterior view]. Pre-operative balloon occlusion test showing the anterior communicating artery and delayed filling of the right ICA territory [**(d)**, anteroposterior view]. Double superficial temporal artery–middle cerebral artery (STA–MCA) bypass was performed **(e)**. Intraoperative angiography showing the good patency of the anastomoses **(f)**. The intra-operative motor evoked potential and somatosensory evoked potential remained stable after temporary occlusion of the ICA for 30 min **(g)**. The ICA was occluded permanently with detachable balloons **(h)**. Controlled angiography showing the good patency of the double STA bypass and the MCA territory that was compensated very well, but the aneurysm was still stasis and fed by the anastomosic artery from the external artery **(i)**. Post-operative perfusion CT showed no ischemic area on the right hemisphere **(j)**. Digital subtraction angiography follow-up revealed the total obliteration of the aneurysms and the good patency of grafts **(k)**.

**Figure 3:**
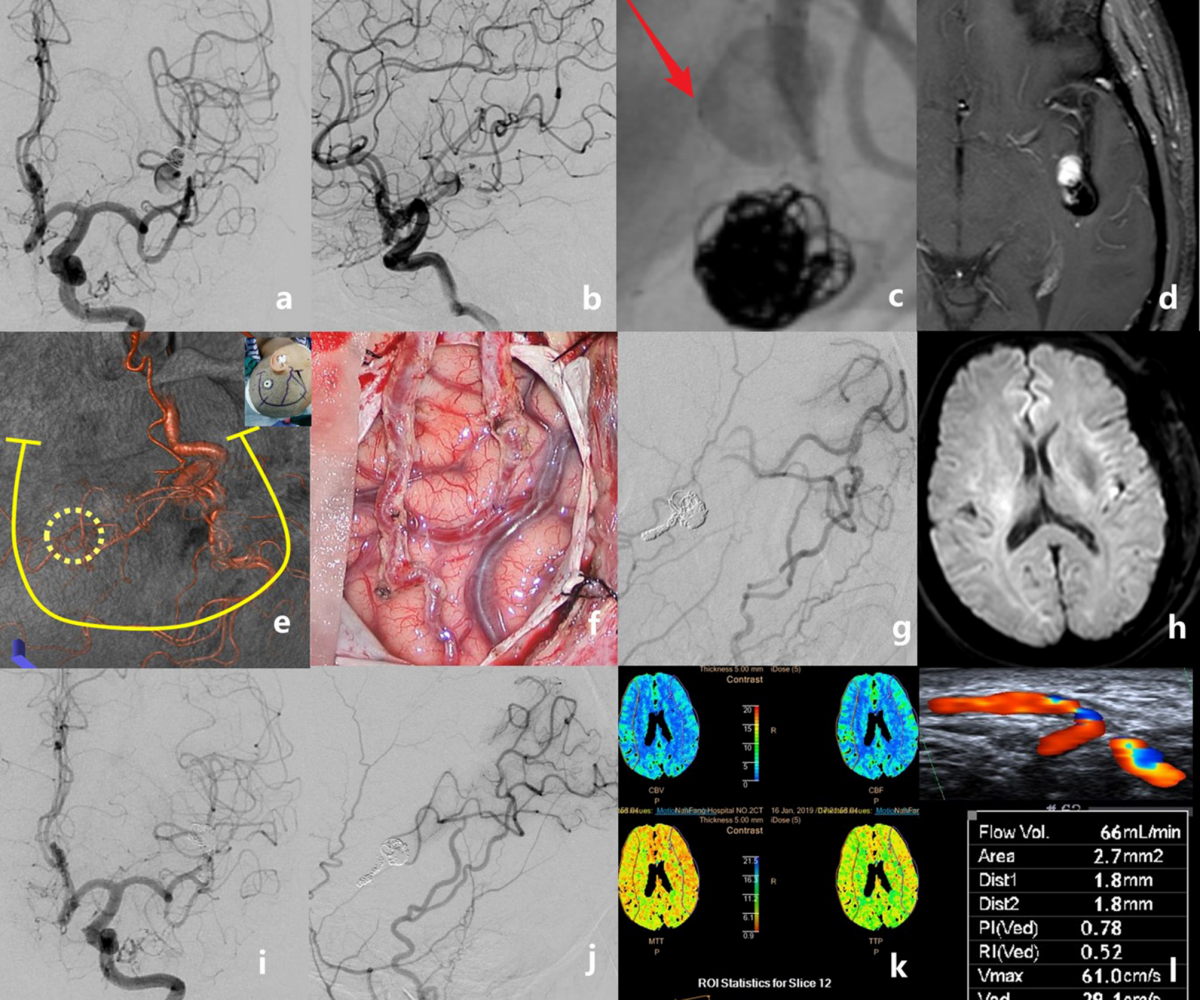
The case of a middle-aged patient with a recurrent middle cerebral artery (MCA) aneurysm after stent-assisted coil embolization is presented. Preoperative angiograms showed that the aneurysm was located at the inferior trunk of the left MCA [**(a)**, anteroposterior view; **(b)**, lateral view]. The stent deformation-related vascular injury developed into a new dissection just proximal to the previous coiled aneurysm **(c)**. Pre-operative MRI showing the intra-aneurysmal enhancement **(d)**. Recipient and donor vessels were precisely marked on the surface of the skull, then a *U*-shaped flap with a small bone window was designed **(e)**. Double superficial temporal artery (STA)–MCA bypass was performed **(f)**. Intraoperative angiography showed the patency of anastomoses; the aneurysm and the parent artery were embolized with coils **(g)**. No new infarct was detected on the post-operative diffusion-weighted imaging **(h)**. Digital subtraction angiography follow-up at 6 months revealed the total obliteration of the aneurysm and the good patency of grafts **(i,j)**. Perfusion CT showed no ischemic area in the bypassed hemisphere **(k)**. The graft flow was 66 ml/min **(l)**.

Intraoperative angiography showed a good patency of all the 38 anastomoses (18 double-barrel anastomosis and two single anastomosis). Neither MEP nor SEP abnormality was detected. After the parent artery and the aneurysm were occluded, the intraoperative angiography showed an immediately successful exclusion of the aneurysm in 20 aneurysms and significant immediate contrast stasis in two, including one MCA aneurysm, which was loosely packed with coils to avoid acute M1 occlusion-related perforator damage. Post-operative CT demonstrated thrombosis in all the 22 aneurysms. Aneurysmal thrombosis was confirmed in all patients by MRI. MRA showed a patency in all the 38 grafts. No surgical site infection was observed after surgery. All patients recovered uneventfully, without ischemic or hemorrhagic complication. When these 20 patients were discharged from the hospital, the Glasgow Outcome Scales (GCS) were grade V in 19 and grade III in one (preoperative GCS was 8), respectively. DSA at 6-month follow-up showed a good graft patency in all the 38 bypasses. One ICA aneurysm recurred with retrograde blood supply from the posterior circulation *via* the posterior communicating artery. The remaining lumen was noted in another MCA aneurysm treated with loose packing. These two aneurysms were retreated by coils through the trans-posterior and trans-anterior communicating artery approach, respectively. The post-operative mean graft flow was 124.2 ml/min (52–227 ml/min), with a significantly dilated diameter which increased from 0.15 cm preoperatively (0.10–0.21 cm) to 0.20 cm postoperatively (0.16–0.27 cm) (Table 2). Representative cases are shown in Figures 2, 3.

**Table 2:** Patients, aneurysms, and treatment characteristics.

**No of patients**	
Female	13
Male	7
Total	20
**Age, year**	
Mean	42.5
Range	8-60
**Aneurysm type**	
Saccular	7
Dissection	3
Serpentine	7
Recurrent after stented	5
**Aneurysm location**	
ICA-cavernous segment	14
ICA-supraclinoid segment	3
MCA-M1 segment	2
MCA-superior trunk	1
MCA-inferior trunk	2
**Bypass type**	
Single STA-MCA	2
Double STA-MCA	18
**Aneurysm treatment strategy**	
Balloon occlusion of ICA	13
Coil embolization of aneurysm and parent artery	4
Balloon occlusion and coil embolization	3
Mortality	0
**Ischemic complication**	
Yes	0
No	20
**Graft flow, ml/min**	
Range	52–227
Mean	124.2
**Aneurysm follow-up**	
Cured	18
Residual and retreatment	2

## Discussion

Giant aneurysm, recurrent aneurysms after stent-assisted embolization, and serpentine aneurysms are rare but typical intracranial complex aneurysms (1–12, 14, 15). The primary goal of treatment is to exclude the aneurysm from the circulation and preserve the normal anatomy and function. These aneurysms have traditionally been treated with aneurysm clipping surgery, with or without thrombectomy. The perioperative morbidity and mortality rates, however, were 30–35% (16). In recent years, the development of interventional treatment, such as the pipeline embolization device (PED), has greatly improved the treatment for giant aneurysm but not for serpentine aneurysm in terms of its high risk of recurrence and technical difficulty (17). The PED is also off-label for recurrent previously stented aneurysms (18). Some patients would refuse to accept PED therapy due to its high cost, so EC–IC bypass combined with parent artery occlusion remains an effective and economic way to treat these complex aneurysms. In our study, 20 patients with 22 complex aneurysms were successfully treated with STA–MCA bypass combined with parent artery endovascular occlusion. All these patients showed a good outcome, indicating that this is an effective way to cure the aneurysm, and much less invasive because there was no need to directly expose and manipulate the complex aneurysm.

In the treatment of cerebral aneurysm that requires parent artery sacrifice, the optimal choice of bypass graft remains controversial (19). The most important issue is to determine the flow demand and assess the adequacy of potential *in situ* donors in order to get a good balance between the supply and the demand. Usually, saphenous vein (SV) or radial artery would be chosen as grafts to introduce generous blood flow from the external carotid artery to the sacrificed territory after the acute ICA occlusion, known as high-flow bypasses. However, if an Allen test is positive, the radial artery cannot be harvested on account of poor palmer collateral circulation (20). The valves are thought to be one of the causes of SV graft failure (21). The risk of bypass graft failure at long-time follow-up was also another issue that should be considered (5–12, 14–21). By comparison, the STA can be harvested easily from the scalp during craniotomy used for the ICA and MCA aneurysm exposure so that the whole surgery is done with only one skin incision. Furthermore, the STA graft has shown a highest long-time patent rate so far. In summary, the STA would be an ideal graft for EC–IC bypass, but the STA–MCA bypass was traditionally considered as a low-flow type graft that may lead to ischemic complications in an acute ICA/MCA therapeutic occlusion. However, recent studies have found that the STA may play a more effective role in bypass surgery than previously thought. In Cherian's study (13), the maximum best observed flow (BOF) with double-barrel bypass was 120 cc/min, and 53% provided BOF ≥65 cc/min. Amin-Hanjani et al. (20) found that flow measurement in the affected vessel at baseline could predict the flow required for full replacement. For MCA aneurysms and proximal ICA aneurysms, the mean required flow was 50 ± 25 and 26 ± 18 cc/min, respectively. In our previous study (11), the mean flow in the STA after bypass was 106.7, 112.6, 97.4, and 79.7 ml/min on post-operative day 1 and day 7 and at 3 and 6 months post-operatively. All these results indicated that an STA bypass has the potential to provide sufficient flow to allow parent artery sacrifice, especially in patients with MCA aneurysms.

However, in patients with ICA aneurysms that need ICA occlusion, the key point for this STA bypass strategy is the presence of well-developed collaterals in the circle of Willis, which can provide a collateral flow in large vessel occlusion conditions. All patients with ICA aneurysms in this study had well-developed anterior and/or posterior communicating artery as confirmed by the BOT angiogram, and in addition, the negative results of the BOT further assure the presence of good collaterals (22, 23). In our hypothesis, the STA–MCA bypass was adequate according to this collateral pattern but cannot be confirmed since the patients were under a general anesthesia during the whole hybrid procedure. Then, intraoperative monitoring of MEP and SEP was used as a real-time evaluation of the bypass function. MEPs had been widely applied in intracranial vascular surgeries in the last decades (24, 25). During parent vessel occlusion, if the flow from the STA graft and communicating arteries is insufficient, it may lead to hypo-perfusion-related infarction; MEP and SEP changes can serve as an early warning, indicating the need for changing the bypass strategy, and the temporary occlusion balloon would be deflated immediately, followed by a rescue bypass with the radial artery or SV. Fortunately, there were no intraoperative MEP or SEP changes during the temporary occlusion in our cases, confirming that the preoperative choice of STA bypass was adequate.

There are several ways to occlude the parent artery after a bypass. In earlier studies, the aneurysms and the parent artery were exposed and surgically clipped or ligated with sutures (26, 27). This procedure required more time and additional incision for ICA ligation. In Mao's study (28), the ICA occlusion was performed by placing the Selverstone clamp around the cervical ICA and gradually clipping it within 7 days. Combined surgical and endovascular treatments of complex cerebrovascular diseases in the hybrid operating room have become popular in the recent years (29–31). However, in a two-stage hybrid treatment, bypass occlusion is a potential risk due to the low-pressure gradient between the EC and the IC systems before the occlusion procedure. Meanwhile, psychological distress increases during the multiple stages of the treatment strategy. Therefore, single-session combined therapy is a better option to avoid these problems, so we introduced a combined strategy to complete the therapy in a hybrid operating theater with good surgical outcome.

This study has two limitations. First, this is a retrospective study with a limited number of patients. Further studies with a larger sample size can help to verify the efficacy and the safety of this mini-invasive hybrid strategy. Second, the patients included in this study were screened by strict preoperative imaging evaluation, which means that this hybrid strategy should be used in certain cases.

## Conclusion

STA–MCA bypass, in combination with endovascular exclusion, is an appropriate option in patients with complex cerebral aneurysms that are not amenable to either surgical clipping or endovascular embolization.

## Data Availability Statement

The original contributions generated for the study are included in the article/supplementary material, further inquiries can be directed to the corresponding author/s.

## Ethics Statement

The studies involving human participants were reviewed and approved by Institutional Review Board of Nanfang Hospital. Written informed consent to participate in this study was provided by the participants' legal guardian/next of kin.

## Author Contributions

All authors listed have made a substantial, direct and intellectual contribution to the work, and approved it for publication.

## Conflict of Interest

The authors declare that the research was conducted in the absence of any commercial or financial relationships that could be construed as a potential conflict of interest.
